# The key to happiness in collaborative workplaces. Evidence from coworking spaces

**DOI:** 10.1007/s11846-022-00558-0

**Published:** 2022-06-01

**Authors:** Domenico Berdicchia, Fulvio Fortezza, Giovanni Masino

**Affiliations:** Department of Economics and Management, University of Ferrara, Via Voltapaletto 11, 44121 Ferrara, Ferrara Italy

**Keywords:** Coworking, Collaborative workplaces, Climate for cooperation, Job crafting, Happiness

## Abstract

This quantitative study explores the significant psychological and behavioral dynamics in coworking spaces. We collected data from a sample of 175 people working in Italian coworking spaces and found that a more cooperative organizational climate increases coworking space users’ happiness. We also found that this relationship is positively moderated by several job crafting behaviors. More specifically, when workers are proactive in the work environment, they are more likely to benefit from the potential advantages (resources, challenges, networking opportunities, etc.) that cooperation-oriented work settings provide, which, in turn, amplifies the positive effect of cooperative work settings on individual happiness. These findings make a useful contribution to both the growing literature on coworking spaces and the more general job crafting literature. Indeed, the previous research on both behavioral dynamics that are specific to coworking spaces and on the role played by job crafting in influencing workers’ happiness remains limited. The study’s managerial implications concern the relevance of establishing a cooperative climate and encouraging workers’ proactivity to promote their happiness.

## Introduction

In the last few years, work organization has changed significantly in many different contexts (Spinuzzi et al. 2018) for several reasons. One such reason is the emergence of the knowledge economy (Clifton et al. 2019; Morisson 2019; Nikolopoulos and Dana 2017; Orel and Kubátová 2019), in which workers are called upon to act as “entrepreneurs and innovators” (Leclercq-Vandelannoitte and Isaac 2016), even when they are wage-employed. In this context, several companies have become increasingly interested in offering knowledge workers alternative (not necessarily internal) workspaces (Brown 2017; Nagy and Lindsay 2018) to better suit their needs and expectations in terms of flexibility, autonomy, knowledge enrichment, and social interactions (Bouncken et al. 2020a). Moreover, an increasing number of start-uppers, freelancers, and independent professionals (Appel-Meulenbroek et al. 2020; Bouncken et al. 2020b; Bouncken and Reuschl 2018; Butcher 2018) have sought innovative solutions to satisfy their need to counter the sense of isolation with which these kinds of workers usually struggle (Blagoev et al. 2019; Gandini 2015; King 2017; Orel et al. 2021), while simultaneously combining their own skills and resources with those offered by other knowledge workers and like-minded people (Bouncken et al. 2018; Garrett et al. 2017).

This has led to a variety of “collaborative workplaces” (Avdikos and Pettas 2021), which represent significant examples of the sharing economy Bouncken et al. 2020b; Merkel 2015; Reuschl et al. 2021). Among the many emerging models and archetypes (Avdikos and Merkel 2020; Bouncken et al. 2018; Resch and Steyaert 2020), independently owned coworking spaces (Appel-Meulenbroek et al. 2020) are receiving a great deal of attention as they offer users unique opportunities to combine autonomy, self-determination, and cooperation (Waters-Lynch and Duff 2021) through an “orchestrated” (Brown 2017) interplay of formality and informality (Bouncken et al. 2020a, b). Coworking spaces portray a new concept of “organizationality” (Appel-Meulenbroek et al. 2020; Blagoev et al. 2019; Butcher 2018; Garrett et al. 2017) based on the following key elements (Bouncken and Reuschl 2018; Bouncken et al. 2021; Bouncken et al. 2022; Brown 2017; Bueno et al. 2018; Gandini 2015; Garrett et al. 2017; Goermar et al. 2021; Merkel 2015; Resch et al. 2020; Rese et al. 2021; Spinuzzi et al. 2018). The first is “openness” since coworking spaces are usually open to all kinds of users (based on an explicit membership) and a wide range of external partners. The second is an “inherent relationality” since the sharing of spaces allows and pushes coworking users to continuously interact, which can be further encouraged by the setting and initiatives promoted by the coworking space providers as “non-neutral agents.” Finally, the third is a “challenging nature” due to both the possibility and need for users to autonomously manage their daily activities (including relational activities) in order to make the most of them.

In this view, coworking spaces can be framed as “dynamic assemblages” (Avdikos and Pettas 2021; Jakonen et al. 2017) where various kinds of users can find ways to develop social ties with inspiring people, learn from them, improve idea generation and implementation (Butcher 2018; Gerdenitsch et al. 2016), and build new business opportunities or creative ideas while actualizing their unique configuration of personal strengths, talents, and preferences (Orel et al. 2021; Spinuzzi et al. 2018). These aspects appear to be consistent with the main drivers of happiness (Deci and Ryan 1985; Nezlek 2001) and particularly with the drivers of happiness at work (Rego et al. 2011), which is an increasingly relevant topic within the managerial and organizational discourse (Liu et al. 2020; Salas-Vallina et al. 2020) as work environments progressively become structured in a bottom-up, loose fashion (Bouncken et al. 2018; Garrett et al. 2017; Spinuzzi 2012). Indeed, perceived happiness is widely conceived as the most powerful factor for positively driving workers’ behaviors (Singh and Aggarwal 2018).

Based on these premises, this study’s first goal is to verify whether a positive relationship exists between the cooperative dimension of coworking spaces (measured through the construct of “climate for cooperation”) and the perceived happiness of coworking users. However, since users’ participation in these new collaborative spaces (Appel-Meulenbroek et al. 2020) requires specific effort (Butcher 2018; Resch and Steyaert 2020) to take advantage of the setting afforded by the providers, any attempt to explore the outcomes of these new forms of collaborative, self-organized work cannot leave aside a contextual analysis of the role played by the specific behaviors initiated by the individuals themselves. It seems plausible that proactive individuals may be more able to take advantage of the opportunities provided by a cooperative environment and, consequently, to enjoy a more significant effect on their happiness. To test this idea, we chose to focus on job crafting (Wrzesniewski and Dutton 2001), which is a specific set of behaviors through which individuals proactively change and re-shape their jobs in various ways. Thus, our study’s second goal is to verify whether job crafting moderates the relationship between a climate for cooperation and happiness.

Overall, we believe that our study contributes to the existing literature on coworking spaces by shedding light on both the organizational and psychological dynamics that make these environments uniquely interesting (Blagoev et al. 2019; Bouncken et al. 2018; Butcher 2018; Garrett et al. 2017; Orel et al. 2021) as a compelling manifestation of a “cultural revolution” occurring in work methods and dynamics (Leclercq-Vandelannoitte and Isaac 2016; Vidaillet and Bousalham 2018; Waters-Lynch and Duff 2021), which is expected to further accelerate due to the ongoing coronavirus pandemic (Mayerhoffer 2021). In the next section, we describe the conceptual background of our study and the rationales for our research hypotheses. The following section explains the study’s methodological approach. The results are then reported, followed by a discussion of the study`s findings, managerial implications, conclusions, and limitations, and possible directions for future research.

## Conceptual background and research hypotheses development

### The key features of coworking spaces as collaborative workplaces

Though the very first prototypes of shared workspaces date back to the mid-90s, the coworking space model began to gain popularity in 2005 in San Francisco and London (Waters-Lynch and Duff 2021) and progressively spread all over the world, with a huge rise in the last few years (King 2017).

According to figures estimated pre-COVID-19, up until 2022, the average annual growth rate of the entire spectrum of shared workspaces (e.g., coworking spaces, hackerspaces, business incubators, accelerators) worldwide was 13% (Appel-Meulenbroek et al. 2020), with around 5 million users (Avdikos and Pettas 2021) of almost 50,000 spaces (Rese et al. 2020), most of which are classified as coworking spaces.

Coworking spaces represent a specific way of organizing daily work activities that is geographically diffused, floating in time, and dispersed in its modalities (Bouncken and Aslam 2019). Such spaces are a reversal of traditional work paradigms (Leclercq-Vandelannoitte and Isaac 2016; Vidaillet and Bousalham 2018), in that they are based on a systematic combination of community participation, individual agency (Butcher 2018), and (partially) “distributed governance” (Resch and Steyaert 2020).

The coworking formula is mostly enacted by independently owned companies that operate from the sharing economy business perspective (Belk et al. 2019) by offering an access-based (Bardhi and Eckhardt 2012) collaborative working experience to different types of users. From this standpoint, the coworking space sector is rapidly evolving by developing increasingly complex and structured value propositions.

For a (usually) monthly fee, the more advanced coworking spaces provide users with both office facilities (e.g., desks, printers, kitchens, lounges) and amenities purely for relaxation and recreation (e.g., napping rooms, small gardens, games rooms, gyms), and even well-being services and social experiences (e.g., meditation classes or parties) (Appel-Meulenbroek et al. 2020). The extended working hours, which often include 24/7 access, also offer users huge flexibility and encourage strong social interactions (e.g., sharing late dinners). This situation blurs the boundaries between work and home and between colleagues and friends (Resch et al. 2020). Consequently, coworking users tend to open themselves up to other users of the space, make friends, and see each other even outside of the workspace.

In wider terms, not only do contemporary coworking spaces provide a rental service of shared tools and facilities, they also put specific effort into designing a powerful “setting” (i.e., the spaces’ environment, rules, restrictions, and options) and conducting daily “curation” processes (e.g., promoting collective routines such as weekly breakfasts or ad hoc events) through their community managers to give users a fruitful, rich, and smooth coworking experience (Bouncken et al. 2020b, 2022; Butcher 2018; Merkel 2015; Rese et al. 2021). In other words, they set the stage for the emergence of a collaborative working experience among “peers” (Bouncken et al. 2020b; Resch et al. 2020), who may have an explicit purpose of social belonging (Garrett et al. 2017) or may discover it along the way.

This is a key organizational aspect of the coworking space model which, though non-hierarchical (Resch and Steyaert 2020), is, at the same time, not “anarchical” despite the presence of “fluid organizational arrangements” that need to be “constantly renegotiated” (Gandini 2015; Jakonen et al. 2017; Vidaillet and Bousalham 2018). Indeed, all the organizational elements (e.g., rituals and routines) established by the coworking space providers pattern the work activities of the space’s users, thus leading to a special “organizationality” (Bouncken et al. 2020a; Butcher 2018) that is “both intentionally created and emerging—formal and informal” (Blagoev et al. 2019, p. 910).

Overall, coworking can be framed as a co-created experience (Bouncken and Tiberius 2021; Garrett et al. 2017; Resch and Steyaert 2020) in which each user has the opportunity to freely choose the role they wish to play within the framework designed by the coworking space provider (Bouncken et al. 2020a; Bouncken and Reuschl 2018; Gerdenitsch et al. 2016). Within this framework, it is left completely to the users to decide whether to interact with others on looser or more intense terms, to be open only in job-related issues or also in private issues, to what extent to receive/provide feedback, and how to fine-tune private and business relationships (Bouncken and Reuschl 2018). In this way, coworking users exercise their agency, in collaboration with others, to participate in social activity and “communal responsibility” (Butcher 2018). This leads to continuous “participatory change” (Kopplin 2020; Resch and Steyaert 2020) and organizing as a set of fluid processes whereby needs and desires are cooperatively formulated and met.

In other words, the extent to which coworking users benefit from their working experience is related both to the “collaborative potential” of each given coworking space and to the users’ ability to craft their daily activities and relational processes in a way that suits their needs and expectations (Bouncken et al. 2018; Leclercq-Vandelannoitte and Isaac 2016; Waters-Lynch and Duff 2021).

Nevertheless, it must be also acknowledged that not all independent coworking spaces have the same “community potential.” As such, we need to separate the so-called “entrepreneurial-driven” coworking spaces (e.g., WeWork) from the more “community-led” spaces (Appel-Meulenbroek et al. 2020). The former manifest themselves as franchised or multi-location coworking spaces (Orel et al. 2021), meaning they are typically larger and more commercially oriented (Blagoev et al. 2019). Such spaces mainly serve startups and tech freelancers/companies, aiming to promote business development. The latter are structured in a more bottom-up manner and are oriented toward independent workers and small and social enterprises that tend to be more interested in the “collaborative use of common pool resources and new, hybrid labour (re)arrangements” (Avdikos and Pettas 2021, p. 44). Consequently, they display, in our view, higher potential as an innovative organizing paradigm, which is why we focus on this kind of coworking space to pursue our research goals.

### The concept of happiness (at work)

Over the last decade, the construct of happiness has come to the fore as a popular topic in several domains and streams of literature. This rising interest in happiness is the result of deep societal changes (Ayala et al. 2017; Mogilner 2019), in which many people are striving to find new balance in several areas of their lives, including work (Stephan et al. 2020). Indeed, within the more fluid and unstable society of the postmodern world, the proactive pursuit of happiness appears to be a top priority for people (Fitriana et al. 2022) who tend to adopt more flexible existential pathways (Van Hugten et al. 2021).

Happiness is a multifaceted construct that stems from a global assessment of one’s life (Fitriana et al. 2022). It is often used as a synonym of well-being (Marescaux et al. 2019) as it concerns individuals’ optimal experience and functioning (Ryan and Deci 2001). Happiness includes aspects of both the hedonic (i.e., pleasure attainment and pain avoidance) and the eudaimonic (i.e., the search for meaning and human growth) views of life (Kahneman et al. 1999; Waterman 1993). The latter represents the most impactful view for most scholars (Peralta and Saldanha 2017; Ryan and Deci 2001), along with warm, trusting, and supportive interpersonal relationships (i.e., relatedness).

Within the wider spectrum of individual happiness, happiness at work plays a significant role given the significant amount of time that people spend at the workplace and the complex array of meanings with which work-related activities and events are imbued (Basinska and Rozkwitalska 2020). In other words, as a substantial part of life, work greatly affects individual happiness, with the two being very intertwined domains. This is even more evident in the disruptive new world that is emerging, where boundaries between work and leisure are progressively blurring (Parker and Grote 2020) and people may start feeling the need or may be required to adopt completely new routines and practices.

From this standpoint, happiness at work is not only a specific driver of productivity and high-quality performance (Salas-Vallina et al. 2020) but also the most important factor in encouraging workers to be more ready and/or willing to face new challenges (Galván Vela et al. 2021).

Happiness at work is an umbrella concept (Salas-Vallina et al. 2020) that relates to both environmental and personal factors Fisher 2010; Singh and Aggarwal 2018; Van Hugten et al. 2021). The former include the structural conditions of the job (i.e., job design) and the organization (e.g., intangible organizational assets, procedures, and rules), as well as discrete or short-lived daily experiences and social interactions that create the basis for people to feel fully satisfied, enriched, and rewarded by what they do. The latter include certain stable attributes of individuals (e.g., personal traits or psychological flexibility), which can foster a happy work life, and their ability to express agency (Tandler et al. 2020).

From this viewpoint, happiness at work should not be framed as something to be taken for granted under “golden” job standards or conditions, but something to be proactively experienced by workers (Blagoev et al. 2019; Bouncken et al. 2020a; Merkel 2015; Oerlemans and Bakker 2018; Waters-Lynch and Duff 2021). This perspective led us to formulate our research hypotheses on the factors and dynamics that can foster happiness in the coworking experience. To this end, we refer to the cooperative dimension of coworking spaces (expressed by the construct of “climate for cooperation”) as a key environmental factor and to “job crafting” as a preeminent form of individual agency.

### The relationship between climate for cooperation and happiness at work

In a seminal attempt to revise the concept, Lu and Argyle (1991, p. 1019) defined cooperation as “acting together, in a coordinated way at work, leisure, or in social relationships, in the pursuit of shared goals, the enjoyment of the joint activity, or simply furthering the relationship.” The authors identified four main classes of cooperation: joint task activity, social relationships, coordination of joint activities, and communication and interaction (Wagner 1995). However, among the organizational literature, studies on cooperation have multiplied, and a variety of concepts and theories have been proposed in different research fields that encapsulate the idea of cooperation (Castañer and Oliveira 2020).

When the focus is on individuals, previous studies have typically investigated the emotions, motivations, and personal preferences that drive cooperation, as well as individual cooperative behaviors (LePine and Van Dyne 2001). For example, Big Five theorists have emphasized the relevance of studying individual personality dimensions (e.g., agreeableness) to better understand cooperative dynamics (Barrick and Mount 1991, 1993). At the same time, the proactivity literature has also explored cooperation through several behavioral constructs, for example, helping behavior, organizational citizenship behavior, altruistic behavior (Grant and Ashford 2008). Here, cooperativeness is conceptualized as a prosocial behavior that is characterized by actions aimed at contributing to the group’s wellbeing and common purpose.

Conversely, when the focus is on the environment, prior studies have investigated the social, organizational, and cultural conditions that represent the context of cooperative processes (Simpson and Willer 2015). The idea of organizational climate (Schneider et al. 2013), which captures the social influences of the organizational environment, has been extensively employed. Specifically, scholars have defined a climate for cooperation “as the organizational norms that emphasize personal effort toward group outcomes or tasks as opposed to individual outcomes” (Collins and Smith 2006, p. 547). Other authors have used the same concept to describe the social and organizational conditions in terms of team spirit, support between members (Fritz and Van Knippenberg 2017), and mutual trust (Bogaert et al. 2012). In modern organizations, where knowledge is a precious resource, a cooperative climate often comprises aspects such as sharing knowledge, information, ideas, and viewpoints (Schreurs et al. 2013). In all contexts, many authors have emphasized the benefits that cooperative processes generate for organizations (Simpson and Willer 2015): When cooperative values are promoted, group or community members are encouraged to share their competences, experiences, and knowledge Bouncken et al. 2018; Bouncken and Aslam 2019; Kopplin 2020). The combination of the varied perspectives and approaches to which people are exposed via social interactions is also facilitated. As highlighted above, coworking spaces provide an extremely fertile environment for building various powerful relations (Avdikos and Pettas 2021), through which interactions and exchanges among different workers are multiplied (Perry-Smith 2006). This sharing “spirit” encourages individuals to cooperate and make efforts on behalf of the group while, at the same time, strengthening the expectations about others doing the same (Bogaert et al. 2012). Cooperation among members facilitates the creation of a work atmosphere characterized by harmony, trust, and collaboration (Collins and Smith 2006), which promotes the sharing of resources, projects, and ideas (Bogaert et al. 2012) and decreases opportunism and selfish behavior (Černe et al. 2014). Overall, a cooperative climate increases the expectation of trust, honesty, mutual aid, supportive behavior (Ding and Chang 2020) and an equal mutual exchange of privileges, insights, and learned lessons (Černe et al. 2014).

Based on these premises, we believe that a positive relationship between climate for cooperation and happiness may exist. Indeed, the relevance of work characteristics and environment in influencing job satisfaction, personal growth, intrinsic work motivation, well-being, and happiness is already well established in the literature (Hackman and Oldham 1976). Autonomy, task significance, feedback from others, interdependence, social support, and interaction may also have a positive effect on happiness (Warr 2017).

Several mechanisms may be invoked to explain the relationship between climate for cooperation and happiness. First, when the working climate is perceived as collaborative, colleagues constitute an important source of close relationships and support (Fritz and van Knippenberg 2017). This also seems to apply to the coworking space domain (Gerdenitsch et al. 2016; Moriset 2014). Research has shown that happiness may be increased by the sense of attachment and group membership and the ability to rely on others’ help that are derived from such a climate (Chan and Lee 2006; Tan et al. 2017). Facilitating camaraderie and trusting relationships between employees, inspired by credibility, respect, appreciation, feedback, honesty, egalitarianism, cooperation, and sharing, is recognized as a fundamental practice for improving the work environment and workers’ happiness (Fisher 2010).

Research has also demonstrated that cooperation and altruistic behavior can evolve as a consequence of repeated interactions due to “social viscosity” (Fowler and Christakis 2010). Halbesleben and Wheeler (2012) observed that higher perceived support from a colleague is positively associated with greater investment in interpersonal citizenship behavior. Since the extant meta-reviews have found that the prosocial behaviors that underlie support (sharing, giving, acts of kindness, etc.) lead to happiness (Curry et al. 2018), it seems plausible that in a cooperative work environment, workers will be more likely to promote sharing, supporting, and helping actions, which will generate increased happiness for them.

Third, the association between climate for cooperation and happiness seems to be consistent with the Job Demands-Resources (JD-R) model and the conservation of resources theory (Bakker and Demerouti 2007). According to the JD-R model, job demands are work features that require sustained physical, cognitive, or emotional effort. Job resources, meanwhile, refer to the physical, social, or organizational aspects of a job that may (a) reduce job demands and the associated physiological and psychological costs; (b) be functional in achieving work goals; or (c) stimulate personal growth, learning, and development. Individuals strive to obtain, retain, and protect their own resources as these are salient factors in gaining new resources and enhancing engagement, flow, and happiness (Bakker and Demerouti 2008). For example, Schaufeli and Van Rhenen (2006) found that quality feedback, learning opportunities, and autonomy at work make employees more enthusiastic and joyful. Xanthopoulou et al. (2008) observed a positive relationship between daily support from colleagues and daily self-efficacy, while Xanthopoulou et al. (2012) indicated that climate for cooperation facilitates the development of personal resources such as self-efficacy, self-esteem, and optimism. Consistent with these results, it seems plausible that available resources in a cooperative work environment may contribute to individual happiness.

Finally, it is possible to reach similar conclusions even if we adopt a eudaimonic view of happiness. Starting from the fundamentals of self-determination theory, Ryan et al. (2006) argued that there is a tight connection between a eudaimonic view of happiness and intrinsic values: The idea is that the requisites for a happy life can be found in the satisfaction of basic psychological needs, (autonomy, competence, and relatedness). Thus, if one follows this perspective, a variety of elements may increase happiness, such as the opportunity of pursuing aspirations that are intrinsically meaningful for the individual, the awareness in volition and goal adoption, the sense of efficacy one has with respect to both internal and external environments, and cultivating deep relationships in social contexts that engender volition and vitality. Based on such assumptions, Reis et al. (2000) empirically demonstrated that happiness increases when the desired activities are performed effectively (perception of self-competence), when goals can be freely chosen consistent with one’s values (perception of autonomy), and when one’s viewpoints and ideas can be shared within an integrated relation (perception of relatedness). Thus, we hypothesized:

#### Hypothesis 1

There is a positive relationship between climate for cooperation and happiness.

### The moderating role of job crafting

A deeper understanding of the circumstances through which a certain work environment may become fertile ground for happiness requires consideration of how individuals choose to navigate such an environment in relation to the available opportunities (Fisher 2010). Indeed, it seems reasonable to assume that an environment with plenty of resources would have a limited effect on happiness if the individual is passive and detached. Conversely, more benefits can be achieved if workers are proactive in acquiring and using the available resources consistent with their goals, aspirations, wishes, and needs (Lyubomirsky et al. 2005). Based on this premise, we hypothesized that job crafting may play a significant role in moderating the relationship between climate for cooperation and happiness.

Wrzesniewski and Dutton (2001) described job crafting as the physical and cognitive changes individuals make in the task or relational boundaries of their work. The authors identified three different types of job crafting behaviors: changing task boundaries, changing relational boundaries, and changing cognitive task boundaries. According to this model, the motivation for job crafting arises from three basic individual needs: the need for control, the need for a positive self-image, and the need for human connection. Job crafting may be facilitated or inhibited by job characteristics (i.e., autonomy, interdependence), general motivational orientations (i.e., intrinsic vs. extrinsic motivations), or work orientation (i.e., viewing work as a job, a career, or a calling). When conceptualized within the JD-R model, job crafting is seen as a set of individual behaviors aimed at developing one’s job resources and changing one’s job demands (Tims and Bakker 2010). Thus, in this framework, job crafting behaviors may aim to increase one’s structural job resources (i.e., responsibility and control, ability to decide on processes and problems, knowledge, competencies and skills, professional development, and opportunities to learn and deploy the full range of one’s capabilities), and/or social job resources (i.e., support from colleagues, help, guidance, and feedback). Furthermore, job crafting behaviors may aim to increase challenging job demands (i.e., new projects and activities that provide opportunities to increase personal and professional growth, learning, and mastery) and/or to decrease hindering job demands (i.e., avoiding those activities that make work mentally or emotionally too intense or too problematic). Meta-reviews have also demonstrated that job crafting increases individual well-being, work engagement, job satisfaction, and psychological and subjective well‐being and reduces the chances of poor well‐being as a result of burnout, job boredom, physical complaints, depression, and job strain (Rudolph et al. 2017; Zhang and Parker 2019).

We believe that job crafting behaviors aimed at increasing one’s resources may amplify the positive relationship between climate for cooperation and happiness since such proactive initiatives, within a cooperative work context like a coworking space, may help individuals multiply and extend the scope of the resources that are available to them, with significant positive effects on personal well-being and happiness. A passive approach would constrain the acquisition of new resources to only those circumstances in which others spontaneously share and offer knowledge and experiences. On the other hand, a proactive approach, such as the one conveyed by the job crafting construct, should significantly increase the opportunities for this type of exchange and enriching experience, especially in a work environment characterized by a positive climate for cooperation.

If we focus on job demands, we argue that a similar amplifying effect of job crafting behaviors on the relationship between climate for cooperation and happiness should also be observed. When workers craft their own demands, they normally try to make their job more interesting and exciting while reducing the least interesting and least exciting elements. Doing a job that allows for the full expression of an individual’s abilities and positive cooperation with others should multiply their sense of competence and autonomy, with clear benefits for their happiness. For example, drawing upon the sustainable happiness model (Lyubomirsky et al. 2005; Peralta and Saldanha 2017) found that when a volitional, self-determined, and self-chosen activity is performed with the help and support of others, the effects on happiness are more significant.

Overall, while a resource-rich work environment may help promote individual happiness, the mere availability of resources may produce limited or even null benefits (Halbesleben and Wheeler 2012) if these resources are not deployed and used in work activities that are perceived as meaningful (Stephan et al. 2020), conducive of personal growth and self-realization, and consistent with the goals and intrinsic needs of individuals. Hence, job crafting behaviors aimed at not only increasing one’s resources but also reshaping one’s work activities to make them more interesting, challenging, and meaningful and less hindering should have a positive effect on the relationship between climate for cooperation and happiness. Thus, we hypothesized:

#### Hypothesis 2

The positive relationship between climate for cooperation and happiness is stronger when (H2a) increasing structural resources, (H2b) increasing social resources, (H2c) increasing challenging demands, and (H2d) decreasing hindering demands is higher.

**Fig. 1:**
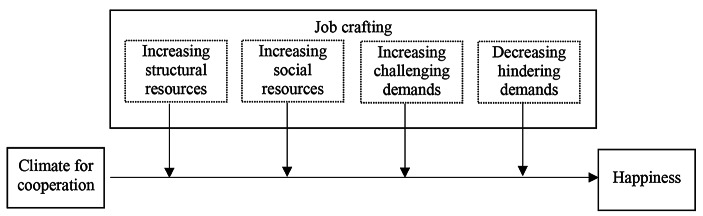
Schematic representation of the model

## Method

### Participants and procedures

The first step of our analysis involved contacting the promoters of the Italian national conference of coworking spaces. This official event gathers together the majority of Italian coworking spaces to share ideas, discuss trends, and find common pathways to enhance the “coworking movement” as a whole. Thanks to the partnership we established with the conference board, we were able to verify our key methodological choices, starting from the sample selection; test and refine our survey questions through a focus session with a significant sample (Creswell and Plano Clark 2011) of coworking users that attended the 2019 edition of the conference; and obtain the list of the Italian coworking spaces that participated in the conference.

Consistent with our research purpose and what the existing literature has revealed, we decided to exclude global entrepreneurial-driven coworking spaces such as WeWork to ensure homogeneity in our sample. To complete the sample, we also searched the Internet by using specific keywords. In this way, we found 12 additional coworking spaces that were not on the list provided by the conference board; thus, we defined a final sample of 239 coworking spaces, which are mostly located in the northern regions of the country (Emilia-Romagna: 15%; Veneto: 10%; Piemonte: 10%; Trentino e Friuli e Liguria: 9%; Lombardia: 14%; Toscana: 13%) and, to a lesser extent, in the southern regions (Lazio: 8%; Umbria e Marche: 5%; Molise, Abruzzo, Basilicata e Puglia: 4%; Campania: 4%; Calabria, Sicilia e Sardegna: 8%).

As we were interested in selecting only coworking spaces with either a formal or “de facto” community manager, we carefully examined each space’s website to ensure sure it fit our sample selection criteria. We contacted the community managers of each of the selected coworking spaces by e-mail and explained the study’s goals to them in detail, as well as the type of coworking space we were interested in and the content of our survey. Ultimately, about 18% of the coworking spaces we approached (42 out of 239) agreed to participate in the study. Each community manager sent the survey to all the coworking members of the community, inviting them to respond to the survey while ensuring their anonymity. We did not find significant differences in the participation rates of coworking spaces according to their regional location.

Overall, we received 190 surveys, but 15 could not be used because incomplete. Our final sample therefore included 175 coworking users (average responses for coworking space = 4; minimum = 3, maximum = 7), 58.3% of whom being female, with an average age of 40.61 years (SD 8.19). 12% have a high school diploma, 58.9% a bachelor degree, 29.1% a master degree or a Phd. These variables do not reveal significant differences among the different coworking spaces. Coworking users were entrepreneurs, consultants, professionals from a wide variety of sectors. Most respondents were web designers (31%), entrepreneurs (23%), writers (7%), management consultants (17%,) project managers (10%), and marketing/sales technicians (12%). About 15% of the marketing/sales technicians and about 12% of all project managers worked steadily for an organization. All the other respondents were self-employed. Considering our respondents’ average age (relatively low, about 40 years old), education level (medium-high), occupation (mostly creative sectors, entrepreneurship, and digital jobs), and self-employment status shows that the general profile seems to be consistent with that found in other previous studies on coworking spaces (Bianchi et al. 2018; Butcher 2018; Rese et al. 2020; Wijngaarden et al. 2020).

### Measures

Since the respondents’ native language was Italian, each measurement scale was translated by a professional translator. We used the back translation method (Brislin et al. 1973) to validate the translation.

#### Climate for cooperation

We adopted a five-item scale developed by Chatman and Flynn (2001) to measure climate for cooperation. To render the scale more appropriate for the context in which we conducted our study, we replaced the term “team members” with “coworkers.” A typical item is “There is/was a high level of sharing between coworkers.” Items were scored on a scale ranging from 1 (strongly disagree) to 7 (strongly agree). The Cronbach’s alpha of this scale is 0.89.

#### Happiness

Happiness was measured through a four item-scale developed by Lyubomirsky and Lepper (1999). A typical item is, “Compared to most of my peers, I consider myself…” scored on a scale ranging from 1 (not a very happy person) to 7 (a very happy person). The Cronbach’s alpha of this scale is 0.89.

#### Job crafting

We measured job crafting through the sub-dimensions of the job crafting scale developed by Tims et al. (2012). The first three dimensions (increasing structural resources, increasing social resources, and increasing challenging demands) include five items each, while the dimension “decreasing hindering demands” comprises six items. In the dimension “increasing social resources,” we eliminated the items that refer to behavior related to seeking support from a supervisor since there are no supervisory roles in coworking spaces.

Some example items for each variable include “I try to develop my capabilities” (increasing structural resources); “I ask colleagues for advice” (increasing social resources); “When an interesting project comes along in my coworking space, I offer myself proactively as project coworker” (increasing challenging demands); and “I make sure that my work is mentally less intense” (decreasing hindering demands). The Cronbach’s alpha estimates for these scales are 0.84, 0.72, 0.85, and 0.74, respectively.

#### Control variables

To rule out the potential confounding effects of socio-demographic variables, we controlled for age, gender, education level, and job tenure. Specifically, participants’ gender and age were used as control variables because they have been suggested to influence people’s subjective level of happiness (Lyubomirsky et al. 2005). We also used education and job tenure as control variables because, as shown in previous studies (Chan and Lee 2006; Salas-Vallina et al. 2018), both may influence available personal and job resources, with different effects on individuals’ ability to develop additional resources and on their happiness. All the scales used are presented in Appendix 1.

## Results

### Preliminary analysis

Before testing our hypotheses, we performed several preliminary analyses. First, since all the data were collected from the same respondents at a single point in time using the same collection method and were self-reported data, we encountered the risk that the results may be biased by common method variance (CMV). Thus, to prevent the potential problems associated with CMV, we took several precautions. We used well-established and reliable scales with varying scale formats. Further, to decrease evaluation apprehension, we also specified that there were no right or wrong answers. We guaranteed respondents’ confidentiality to reduce socially desirable responses (Podsakoff et al. 2012). Finally, our model hypothesized a relationship between the dependent and predictor variable that is moderated by another variable, and respondents are unlikely to recognize “difficult-to-visualize” interaction effects (Chang et al. 2010).

We examined the common method bias through two procedures. First, using AMOS, we examined the common method bias by constructing a common latent factor (CLF) for all the items. We then compared the model with the CLF to the model minus the CLF by using standardized regression weights (Podsakoff et al. 2003). We used a difference of 0.20 to indicate the possible CLF influence. We found no significant difference, suggesting that common method bias was not a significant problem in this sample. Nonetheless, we also performed Harman’s one-factor test (with SPSS) to empirically address the common-method variance issue, and the results confirmed that common-method bias was not a problem in our case.

Second, before testing our hypotheses, we studied the structural validity of the scales used by performing a confirmatory factor analysis in AMOS. The hypothesized six-factor model (climate for cooperation, increasing structural resources, increasing social resources, increasing challenging demands, decreasing hindering demands, and happiness) exhibited an acceptable fit [χ^2^ = 603.39 (df = 309), χ^2^/df = 1.95, CFI = 0.92, IFI = 0.92, TLI = 0.91, RMSEA = 0.06]. We compared the hypothesized model with several other models, although none of them produced a better fit than the hypothesized model: a five-factor model factor model in which increasing structural resources and increasing social resources were combined into one factor [χ^2^ = 664.88 (df = 314), χ^2^/df = 2.12, CFI = 0.90, IFI = 0.90, TLI = 0.88, RMSEA = 0.07] and a three-factor model in which all job crafting variables were combined into one factor [χ^2^ = 1230.26 (df = 321), χ^2^/df = 3.83, CFI = 0.68, IFI = 0.68, TLI = 0.65, RMSEA = 0.13]. We also tested the single-factor model with all the items loaded on a common factor, which showed a very poor fit [χ^2^ = 1948.12 (df = 324), χ^2^/df = 6.01, CFI = 0.37, IFI = 0.38, TLI = 0.31, RMSEA = 0.18].

Next, we calculated the composite construct reliability (CR) coefficients and average variance extracted (AVE) (Table 1). The CR ranged from 0.71 (for increasing social resources) to 0.90 (for happiness), which were above the 0.70 cut-off value; the AVE, which ranged from 0.51 (for decreasing hindering demands) to 0.70 (for happiness), also surpassed the recommended benchmark of 0.50 (Fornell and Larcker 1981). A comparison of the square root of the AVE of each construct with their correlation estimates showed that the square root of the AVE for each dimension was higher than the corresponding inter-construct correlation estimate (Fornell and Larcker 1981). Thus, our model had a satisfactory degree of convergent and discriminant validity. In addition, we examined the alpha coefficients of each scale. All values were greater than 0.70, indicating good reliability.

Finally, because some employees were nested within the same coworking space, we sought to verify that our observations could be treated independently. First, we calculated two kinds of intra-class correlation coefficients (ICC): ICC(1) and ICC(2) (Shrout and Fleiss 1979). ICC(1) is an estimate of the degree to which workers belonging to the higher-level unit (i.e., the same coworking space) responded similarly. According to Hox (2002), coefficients of 0.05–0.09 indicate a low effect, coefficients of 0.10–0.14 represent a moderate effect, and coefficients of 0.15 and above indicate a large effect. ICC(2) is an indicator of inter-rater reliability. ICC(1) ranged from 0.01 to 0.04, while the ICC(2) values for all variables were lower than the 0.60 cutoff point recommended by Glick (1985).

Further, we employed the within-group agreement index of multiple-item scales developed by James et al. (1993). The inter-rater agreement index (rWG(j)) describes the degree to which different raters provide a similar rating for the same stimulus. Values greater than 0.70 have been recognized as representing sufficient response consistency to justify aggregating individual responses to the group level (Klein et al. 2000). In our analysis, all the rWG(j) values were below the critical cut-off value of 0.70.

### Descriptive statistics

Table 1 shows the means, standard deviations, and bivariate correlations among the studied variables and AVE (in parentheses) and provides preliminary support for research hypotheses H1 since climate for cooperation was positively correlated with happiness (r = 0.30, p < 0.001).

**Table 1:** Descriptive statistics and intercorrelations of the variables

	Variables	Mean	SD	Cronbach’s alpha	CR^a^	AVE^b^	1	2	3	4	5	6	7	8	9	10
1.	Age	40.61	8.19	–	–	–	–									
2.	Gender	0.43	0.50	–	–	–	0.11	–								
3.	Job Tenure	9.83	7.30	–	–	–	0.62***	-0.06	–							
4.	Education	3.17	0.62	–	–	–	-0.17*	-0.01	-0.37***	–						
5.	Climate for cooperation	4.49	1.28	0.89	0.89	0.63	-0.04	-0.01	-0.13	0.16*	(0.79)					
6.	Happiness	5.13	1.22	0.89	0.90	0.70	0.11	-0.10	0.18*	-0.14	0.30***	(0.84)				
7.	Increasing structural resources	4.20	0.61	0.84	0.88	0.59	0.06	-0.04	0.01	-0.19*	0.26***	0.36***	(0.77)			
8.	Increasing social resources	3.46	0.85	0.72	0.71	0.55	− 0.27***	-0.11	-0.21**	0.12	0.46***	0.16*	0.30***	(0.74)		
9.	Increasing challenging demands	3.43	0.90	0.85	0.88	0.60	0.01	-0.06	0.14	-0.16*	0.35***	0.12	0.31***	0.55***	(0.77)	
10.	Decreasing hindering demands	2.51	0.65	0.74	0.86	0.51	-0.07	-0.03	0.02	-0.08	-0.7	0.11	0.10	0.10	0.04	(0.71)

N = 175. Values in parentheses display the square root of the average variance extracted. ^a^Composite reliability. ^b^Average variance extracted.

Gender: male = 1; female = 0. Education: 1 = middle school diploma or less; 2 = high school diploma; 3 = bachelor degree 4 = master degree or more.

* p < 0.05;

** p < 0.01;

*** p < 0.001.

### Hypothesis testing

Consistent with previous studies that have examined the moderating function of job crafting, separate hierarchical regression analyses were conducted for each job crafting dimension (Van Hooff and Van Hooft 2014). Hence, we tested four different models. In the first step (which was the same for all the models), we entered age, gender, job tenure, and education as control variables. None of these variables were significantly correlated to happiness. In the second step, we added the independent variable and the job crafting dimension under study. The results (Table 2) showed that climate for cooperation positively influenced happiness in each of the tested models (β = 0.26, p < 0.001; β = 0.31, p < 0.001: β = 0.36, p < 0.001; and β = 0.34, p < 0.001, respectively).

**Table 2:** Results of regression analysis

			Model 1 (DV = happiness) Increasing structural resources as moderator			Model 2 (DV = happiness) Increasing social resources as moderator			Model 3 (DV = happiness) Increasing challenging demands as moderator			Model 4 (DV = happiness) Decreasing hindering demands as moderator	
	Step 1		Step 2	Step 3		Step 2	Step 3		Step 2	Step 3		Step 2	Step 3
Age	0.01		-0.01	-0.01		0.01	0.01		-0.01	0.01		0.01	0.01
Gender	-0.23		-0.20	-0.16		-0.22	-0.17		-0.24	-0.16		-0.23	-0.22
Tenure	0.02		0.03*	0.03*		0.03	0.02		0.03*	0.02		0.03	0.03
Education	-0.18		-0.13	-0.11		-0.27	-0.21		-0.29*	-0.34*		-0.25	-0.25
Climate for cooperation			0.26***	0.22**		0.31***	0.30***		0.36***	0.34***		0.34***	0.34***
Job crafting			0.54***	0.75***		0.07	0.10		-0.09	-0.07		0.23	0.17
Climate for cooperation x Job crafting				0.35**			0.24**			0.26***			0.11
R^2^	0.05		0.23	0.28		0.18	0.21		0.17	0.25		0.18	0.18
ΔR^2^			0.18	0.05		0.13	0.03		1.12	1.08		0.13	0.00
*F*	2.20		8.41***	9.08***		5.63***	6.17***		5.71***	8.04***		6.16***	5.39***

N = 175.

* p < 0.05;

** p < 0.01;

*** p < 0.001.

**Fig. 2:**
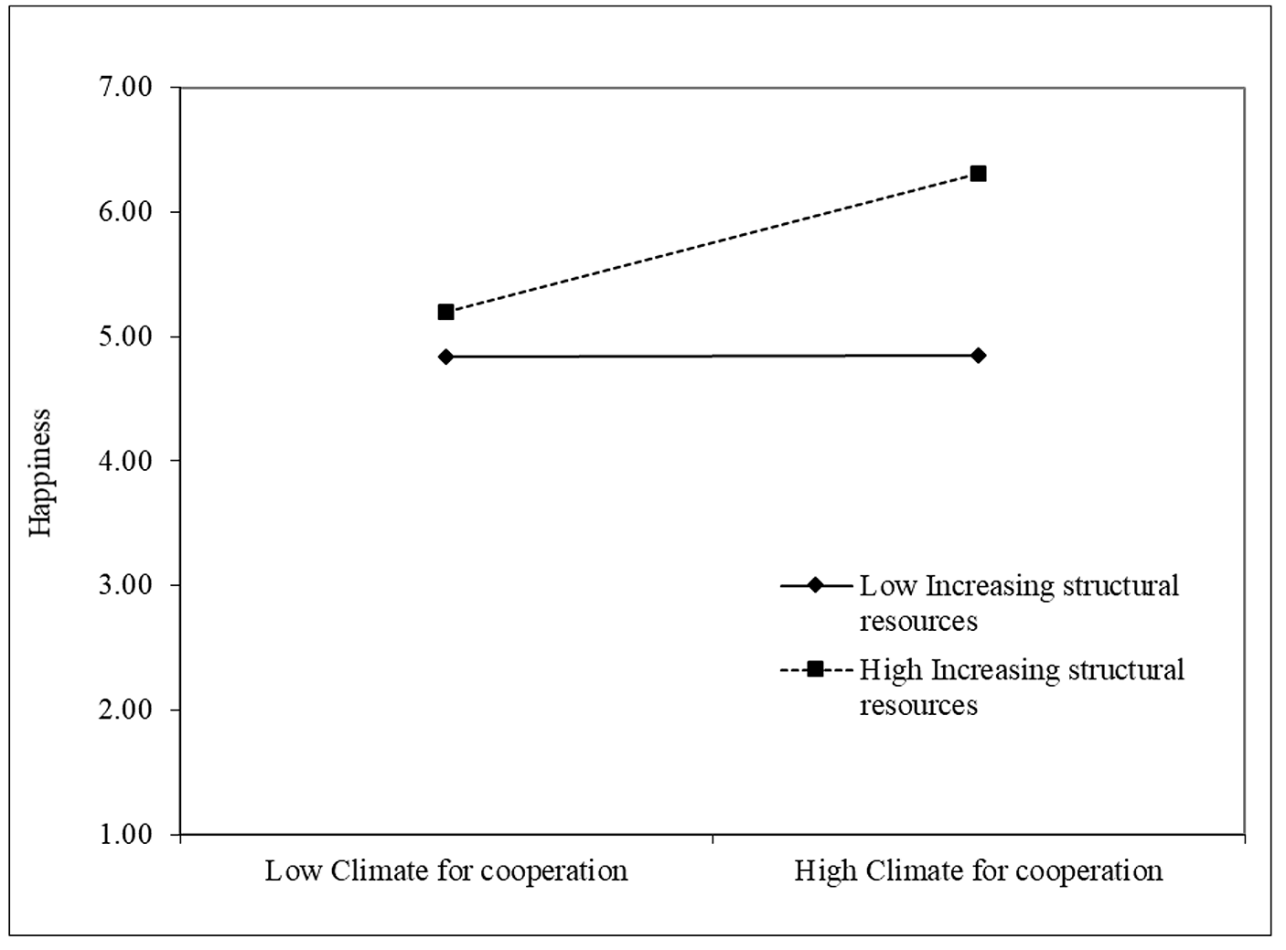
Increasing structural resources as moderator of the relationship between Climate for cooperation and Happiness

**Fig. 3:**
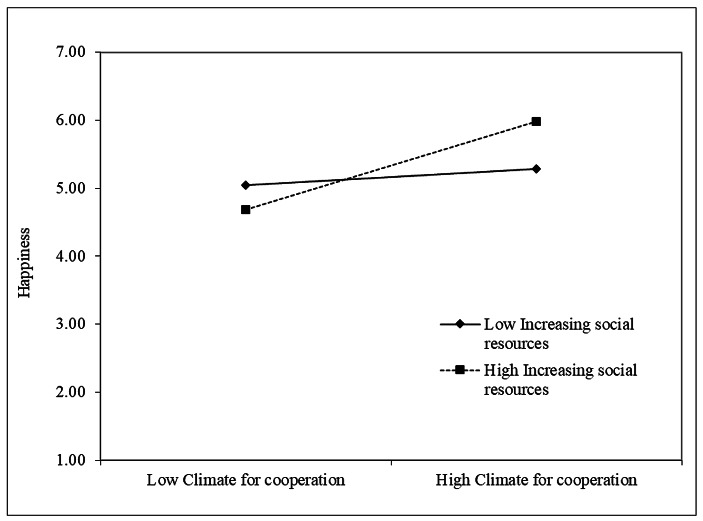
Increasing social resources as moderator of the relationship between Climate for cooperation and Happiness

**Fig. 4:**
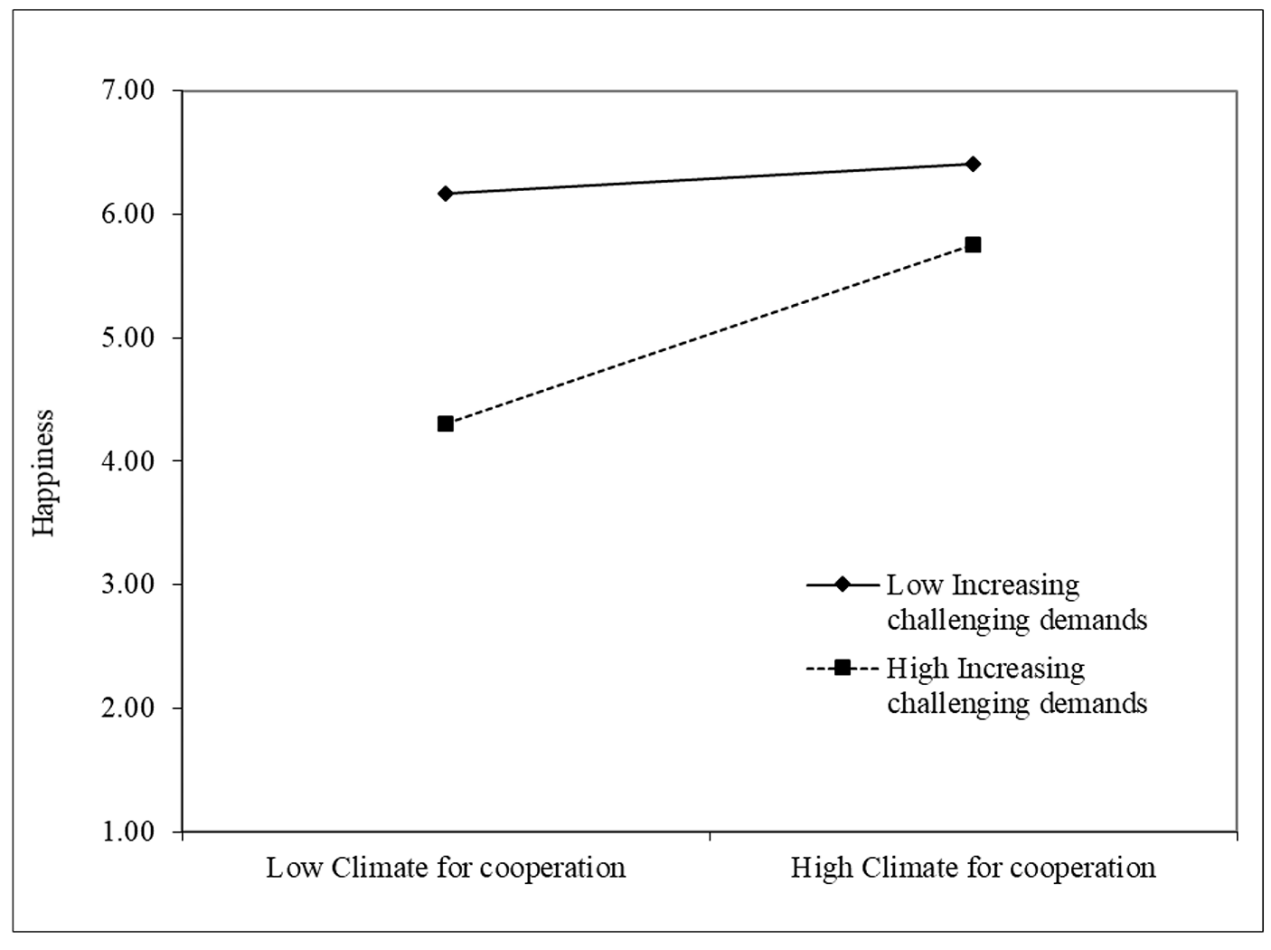
Increasing challenging demands as moderator of the relationship between Climate for cooperation and Happiness

Thus, Hypothesis 1 was supported. Increasing structural resources was the only job crafting behavior that was significantly positively correlated with happiness (β = 0.54, p < 0.001). Finally, in Step 3 of the regression, we added the interaction terms. The relationship between climate for cooperation and happiness was stronger when increasing structural resources (β = 0.35, p < 0.01), increasing social resources (β = 0.24, p < 0.01), and increasing challenging demands (β = 0.26, p < 0.001) were higher. Thus, Hypotheses H2a, H2b, and H2c were supported. Contrary to our expectations, decreasing hindering demands did not moderate the relationship between climate for cooperation and happiness. To provide a clearer representation of the significant interaction effects, we plotted simple slopes one standard deviation below and one above the mean of the climate for cooperation measure (Figs. 2, 3 and 4).

## Discussion

Our study is an early attempt to shed light on the increasing, widespread success of coworking spaces, based on a combination of psychological and organizational dynamics. The growth of this phenomenon requires a closer look at what happens inside coworking spaces in terms of both processes and outcomes. We tested a model focused on two key elements of typical work processes in coworking spaces: on the one hand, the collaborative atmosphere and culture, and on the other hand, the non-hierarchical, autonomous nature of work activities (Bouncken et al. 2020a).

First, we found a positive relationship between climate for cooperation and happiness. In other words, our study suggests that coworking users are happier in coworking spaces that promote cooperation. This result supports some of the key ideas that triggered the diffusion of coworking spaces, which have been conceptualized as organizational solutions that not only provide economic benefits but that can combat the frustration, feelings of being adrift, alienation, and dissatisfaction related to solitude and isolation from one’s professional community (Gerdenitsch et al. 2016). Our result is also consistent with previous related studies. For example, Orel and Kubátová (2019) conceptualized coworking as an emerging integral model of conscious business through which, beyond financial performance, fundamental human needs and a sense of purpose at work can be preserved and satisfied. Similarly, in a qualitative, single case study, Garrett et al. (2017) observed that the most significant benefits of coworking spaces in terms of well-being and psychological health relate to the sense of community and opportunity to satisfy human needs such as affiliation and affection that they provide.

We also found that several job crafting behaviors positively moderate the relationship between climate for cooperation and happiness. This is an interesting result because we believe that to extend our knowledge of the co-working phenomenon and, more generally, of happiness at work, it is important to carefully consider the behavioral and psychological dynamics that coworking spaces seem to trigger. Indeed, such dynamics may be helpful to more satisfactorily explain the processes (knowledge creation, innovation, etc.) upon which the current literature on coworking spaces has mostly focused. More specifically, our results on the moderating role of job crafting behaviors provide a possible explanation of *why* and *how* a collaborative climate may generate happiness. While the literature has mostly focused on the structural/organizational conditions that may facilitate or inhibit the sharing dynamics and outcomes in coworking spaces, the role that coworking users may play through proactive behaviors has been completely neglected. Our study aims to contribute to the existing literature by providing some initial evidence on the impacts of proactivity and job crafting in coworking spaces.

First, we found that what are commonly called “approach” job crafting behaviors (behaviors aimed at increasing job resources and challenging demands) positively moderate the relationship between the collaborative climate and happiness. In other words, when coworkers’ attitudes are not to “passively” take advantage of the opportunities that a collaborative work setting provides but to “customize” and even generate such opportunities in a proactive, personal way, consistent with their needs, aspirations, and inclinations, a positive effect on their happiness can be observed. This result is in line with classic literature on the relationship between autonomy and intrinsic motivation (Deci and Ryan 1985), which has shown through a vast number of empirical studies that autonomy is a key ingredient for satisfactory, intrinsically motivating work experiences. Thus, while coworking spaces seem to provide an ideal setting that is rich in opportunities for cooperation and, indirectly, for increasing available resources and stimulating challenges, it is only when workers craft their jobs in an autonomous, proactive, personalized manner that the real potential for satisfaction and happiness is fully realized. It is worth noting that it seems at least plausible that the same general principle might hold true in other, more traditional organizations and work settings.

While we found that three out of the four job crafting behaviors studied positively moderate the relationship between climate for cooperation and happiness, we did not find the same effect for the job crafting behavior of “decreasing hindering demands.” This fourth type of job crafting behavior is significantly different in nature from the others: While job crafting behaviors aimed at increasing resources and challenging demands involve a “positive” approach to job crafting (i.e., one in which workers try to extend the scope of their jobs), a behavior such as “decreasing hindering job demands” has a “negative” nature, whereby workers try to reduce or avoid specific tasks (Zhang and Parker 2019). Thus, it seems plausible that a very different effect would be observed. As such, in trying to understand the reason why Hypothesis 2d was not supported, we can speculate that the reduction of hindering job demands, while allowing individuals to convey the resources developed through cooperation toward more desirable activities (as we hypothesized), may also decrease the need for extra resources (even though they are perceived as available within the work context), thereby making them less necessary and, consequently, less important for personal well-being and happiness. In other words, alleviating the workload by reducing one’s hindering job demands may reduce the number of problems and difficulties for which the cooperation of coworking users is necessary, thereby reducing the motivation to seek the proximal support that is available within the work context. This may have non-positive or even negative consequences on happiness, which may compensate for the positive effect that we hypothesized. Thus, it seems plausible that the combined effect of these two opposing phenomena is non-significant.

### Managerial implications

Our findings have several managerial implications at various levels. As far as coworking spaces are concerned, we suggest that coworking managers and community managers should focus on augmenting the cooperative climate of their coworking spaces in order to increase coworkers’ happiness, which would certainly improve the coworking spaces’ reputations, attractiveness, and success. While a highly cooperative climate seems to naturally emerge in coworking spaces, managers may nonetheless further increase this cooperation through specific initiatives that promote interaction and cooperation between coworkers. Furthermore, the key importance of coworkers’ proactivity (through job crafting behaviors), as suggested by our findings, implies that managerial initiatives aimed at encouraging proactivity may play a crucial role in generating coworker happiness. The job crafting literature suggests that contextual elements may facilitate or hinder job crafting behavior (Wrzesniewski and Dutton 2001); thus, coworking managers clearly play an important role not only by providing specific opportunities for cooperation but also by encouraging coworkers to proactively explore such opportunities and even create new ones.

At a more general level, one could hypothesize that similar results may hold true in traditional companies. Presently, this remains a hypothesis that could be tested in future studies. However, if this hypothesis were confirmed, it would suggest even more significant implications in terms of the key role played by leaders and human resource managers in trying to develop a cooperative climate and encourage job crafting in their organizations, especially if we consider the intrinsic value of human happiness and, more pragmatically, the positive relationship between happiness and productivity (Galván Vela et al. 2021; Salas-Vallina et al. 2020).

### Limitations and future research directions

Our results should be interpreted in the context of some limitations, which open new opportunities for future research. First, our self-reported cross-sectional data did not allow us to establish with certainty the causal directions of the observed relationships and exposed the results to common method biases. However, our model was based on well-established theoretical assumptions, consistent with the extant literature. We also used several procedural and statistical remedies to reduce the possibility of common method bias. Regardless, it has been established that interaction effects cannot be caused by CMV (Siemsen et al. 2010). Moderating effects are incompatible with CMV, which tends to deflate interaction effects, making their statistical detection difficult. All these elements provide good reasons to have confidence in our findings. To expand our results, future studies may adopt a longitudinal research design or use data from a variety of sources.

Another limitation relates to the particular context that we chose (mostly freelancers operating in coworking spaces) which, on the one hand, may provide unique interpretative opportunities in relation to our study’s goals but, on the other hand, may be somewhat limiting. Indeed, one may wonder about the nature of work activities in coworking spaces and whether they can be conceptualized as job crafting since there is no formal managerial job design. However, the concept of job crafting, as originally proposed in the seminal contribution by Wrzesniewski and Dutton (2001), encapsulates the idea that workers may become “architects” of their own jobs. This idea is strengthened, although differently, within the JD-R model (Tims and Bakker 2010), in which job crafting relates to behaviors aimed at reshaping one’s job resources and demands. Thus, if job crafting describes a process through which individuals re-shape their jobs in relation to their own preferences, goals, and abilities, then coworking spaces represent an ideal context in which such processes may be studied. Indeed, in coworking spaces, it is possible to observe some workers that limit their work activities to fundamental aspects while waiting for customers or colleagues to propose interesting projects, while others are constantly and actively committed to searching for new opportunities and challenging goals and demands. Similarly, some coworking users consistently try to build new networks and extend their social relations as much as possible (in other words, they try to increase their social resources), while others see coworking spaces as just another place to work with marginal benefits. Consistent with our view, the literature has shown that the job crafting research is not limited to workers with formally prescribed positions but can be extended to professionals, self-employed workers, and entrepreneurs (Bredehöft et al. 2015). As such, future studies may explore the relationships that we studied herein among different groups of workers.

While our specific research setting was coworking spaces, future research may explore whether the same results hold true in traditional companies and workplaces. We believe that this is likely the case because though co-working spaces have specific features that create an ideal context for encouraging and facilitating cooperation and proactivity, there is nothing preventing traditional workplaces from providing similar opportunities to their employees in various ways and to different degrees. We believe that the positive relationship between climate for cooperation and happiness, moderated by proactive behaviors such as job crafting, is likely a general phenomenon, which can be positively triggered by favorable work arrangements and circumstances (such as coworking spaces) or constrained and limited in other work situations, depending on the availability of cooperation opportunities. Thus, future research may explore in more detail the significance of contextual variables in different kinds of workplaces and organizational cultures.

A possible limitation of our study concerns the research setting. By utilizing heterogeneous sources, we included some of the most representative coworking spaces in Italy in our study. We are fairly certain that our results are relevant for the Italian context. However, cultural elements may have influenced these results; therefore, caution should be exercised in generalizing them to other national cultures. Such cultural differences could be explored in future studies.

Finally, our study was conducted before the outbreak of the COVID-19 pandemic. Consequently, our data and results refer to a “normal” (pre-pandemic) situation, where physical proximity represents an extremely relevant aspect of work in coworking spaces. Future research may attempt to understand whether the relationship between climate for cooperation and happiness still holds true in work situations in which remote working is prevalent or in which social distancing significantly constrains the possibility for direct interaction and collaboration. We believe that, in their essence, our results should have a general quality as they could hold true not only in coworking spaces but also in traditional workplaces (as long as some opportunities for cooperation are provided and human interactions are allowed to happen in “normal” circumstances). However, it is clear that the very meaning of “climate for cooperation” may change significantly depending on the modalities and tools through which people may (or may not) establish cooperative relations. Co-working itself may indeed evolve in ways that are hard to predict within a post-pandemic world. A reflection on this topic clearly goes well beyond the scope of our study. Nevertheless, we believe that our results may inspire future research to explore if and how constrained modalities of interaction and communication may change the relationships between cooperation, job crafting, and happiness that we have illustrated.

## Conclusions

In summary, this study indicates a positive relationship between climate for cooperation and happiness in coworking spaces. It also shows that this relationship is stronger when coworkers act as job crafters, especially when they proactively reshape their work by increasing their job resources and challenging job demands.

We believe that this line of research on behavioral dynamics in coworking spaces is important for several reasons. First, most previous studies on coworking spaces are either theoretical or conceptual in nature. With some exceptions (Avila et al. 2018; Bouncken et al. 2020a; Bueno et al. 2018; Gerdenitsch et al. 2016; Rese et al. 2020), the empirical evidence that has been presented is either “semi-scientific,” derived from internet blogs (as highlighted by Bouncken and Reuschl (2018, p. 330)), or has emerged from qualitative studies (Clifton et al. 2019). Thus, while studies based on ethnographic and grounded theory techniques, sociocultural approaches, and relational constructionist perspectives clearly suggest that the coworking collaborative climate may have positive consequences for coworking users (Butcher 2018; Houghton et al. 2018; Spinuzzi 2012; Tremblay and Scaillerez 2020; Wijngaarden et al. 2020), this idea has not been tested yet through a quantitative approach. Our study is a first step toward filling this knowledge gap.

Second, our study is one of the first to focus on happiness as a relevant outcome of work in coworking spaces. Coworking is a relatively new phenomenon and, thus far, much attention has been devoted to knowledge creation (Bouncken and Aslam 2019), innovation, and business opportunities (Clifton et al. 2019): As Jakonen et al. (2017, p. 235) stated, “the ideological discourse on coworking is based on an open coworking movement that highlights entrepreneurship and emphasizes how innovation is driven by collaborative practices.” While these elements are certainly extremely important, there is currently a lack of attention to psychological and behavioral dynamics that help explain how and why high levels of innovation and knowledge creation may happen in coworking spaces. Our study is a contribution to this line of research.

Overall, we believe that this line of research should be extended, not only due to the inherent interest in coworking spaces as a relatively new organizational phenomenon but also because increasing our knowledge on the internal dynamics of these new organizational contexts may also inform new research and reflection on more traditional companies (Blagoev et al. 2019; Bouncken et al. 2018, 2020b; Butcher 2018). In traditional work environments, important organizational innovation may be inspired by the experiences and outcomes that we observe in coworking spaces.

## Appendix 1

**Climate for cooperation**.

1. It is important for us to maintain harmony within our coworking space.
2. There is little collaboration among coworkers. Tasks are individually delineated.
3. There is a high level of cooperation between coworkers.
4. Coworkers are willing to sacrifice their self-interest for the benefit of other coworkers.
5. There is a high level of sharing between coworkers.

**Happiness**.

In general, I consider myself:

1. Not a very happy person – (7) A very happy person.

Compared to most of my peers, I consider myself:

1. Less happy – (7) More happy.

Some people are generally very happy. They enjoy life regardless of what is going on, getting the most out of everything. To what extent does this characterization describe you?

1. Not at all – (7) A great deal.

Some people are generally not very happy. Although they are not depressed, they never seem as happy as they might be. To what extend does this characterization describe you?

1. Not at all – (7) A great deal.

**Job crafting**.

**Increasing structural resources**.

1. I try to develop my capabilities.
2. I try to develop myself professionally.
3. I try to learn new things at work.
4. I make sure that I use my capacities to the fullest.
5. I decide on my own how I do things.

**Increasing social resources**.

6. I ask others for feedback on my job performance.
7. I ask colleagues for advice.

**Increasing challenging job demands**.

8. When an interesting project comes along in my coworking space, I offer myself proactively as project coworker.
9. If there are new developments in my coworking space, I am one of the first to learn about them and try them out.
10. When there is not much to do in my job, I see it as a chance to start new projects related to my coworking space.
11. I regularly take on extra tasks during my activity as a coworker, even though I do not receive extra pay for them.
12. I try to make my work more challenging by examining the underlying relationships between different aspects of my job.

**Decreasing hindering job demands**.

13. I make sure that my work is mentally less intense.
14. I try to ensure that my work is emotionally less intense.
15. I manage my work so that I can minimize contact with people whose problems affect me emotionally.
16. I organize my work so as to minimize contact with people whose expectations are unrealistic.
17. I try to ensure that I do not have to make many difficult decisions at work.
18. I organize my work in such a way to make sure that I do not have to concentrate for too long a period at once.
